# The Prevalence and Risk Factors of Iron Deficiency Anemia Among Pregnant Women in Malaysia: A Systematic Review

**DOI:** 10.3389/fnut.2022.847693

**Published:** 2022-04-15

**Authors:** Raudah Abd Rahman, Idayu Badilla Idris, Zaleha Md Isa, Rahana Abdul Rahman, Zaleha Abdullah Mahdy

**Affiliations:** ^1^Public Health Division, Health Department of Wilayah Persekutuan Kuala Lumpur & Putrajaya, Kuala Lumpur, Malaysia; ^2^Department of Community Health, Faculty of Medicine, Universiti Kebangsaan Malaysia, Bandar Tun Razak, Malaysia; ^3^Department of Obstetrics and Gynaecology, Faculty of Medicine, Universiti Kebangsaan Malaysia, Bandar Tun Razak, Malaysia

**Keywords:** serum ferritin, hemoglobin, nutritional deficiency, determinants, compliance, anemia, iron deficiency

## Abstract

Anemia in pregnancy is defined as a hemoglobin level of <11 g/dl, and is commonly due to iron deficiency. This systematic review was conducted to determine the prevalence and risk factors of anemia and iron deficiency among pregnant women in Malaysia. A systematic literature search was conducted in Google Scholar, PubMed, and Cochrane Library databases. We followed the Preferred Reporting Items for Systematic Reviews and Meta-Analysis (PRISMA) guideline. Eight studies comprising a total number of 2,638 pregnant women were included in this review. Only two studies focused on iron deficiency, whereas the other six investigated anemia in pregnancy without specifying iron deficiency or any other nutritional cause for the anemia, signifying the lack of published literature on this important public health nutritional issue in Malaysia. The overall prevalence of anemia in pregnancy ranged from 19.3 to 57.4%, while the prevalence of iron deficiency was 31.6 to 34.6%. Factors that were significantly associated with anemia in pregnancy were extremes of reproductive age, late antenatal booking, non-compliance to hematinics, Indian ethnicity, being in the second or third trimester, low maternal educational level, low family income, and unemployment. The prevalence of anemia in pregnancy was found to be higher in rural compared to urban areas. Meanwhile, in terms of iron deficiency anemia, grandmultiparity, late antenatal booking and Indian ethnicity were significant determinants. It is certainly plausible that the anemia in pregnancy reported in these studies is not entirely secondary to iron deficiency and may be attributable to other nutritional deficiencies, emphasizing the importance of researching deeper into this subject. Nevertheless, in the meantime, focusing on iron supplementation in high-risk mothers with emphasis on compliance, seems to be the best option, in view of the high prevalence of iron deficiency found in this review.

## Introduction

Anemia in pregnancy is defined as a hemoglobin level of <11 g/dl (1). It is further classified into mild, moderate and severe, when the hemoglobin level is between 10.0 to 10.9, 7.0 to 9.9, or <7.0 g/dl, respectively (2). The prevalence of anemia in pregnancy has been reported as 29.9% globally (3). Despite efforts to reduce the incidence of anemia especially in developing countries (4), it is still widespread globally resulting in major health consequences if not adequately managed (5).

Maternal anemia has significant adverse effects on both mothers and infants (6). The risk of death among pregnant women with severe anemia has been reported to be twice that of mothers without severe anemia (7). This is contributed by severe hemorrhage at delivery or postpartum (8, 9) as well as complications due to anemia such as heart failure (8, 10). In terms of fetal outcome, anemia increases the occurrence of perinatal morbidities such as low birth weight, prematurity due to spontaneous preterm birth (9, 11), and neonatal iron deficiency (12, 13).

The World Health Organization (WHO) classifies anemia in pregnancy based on its prevalence. A prevalence below 4.9% is considered as an insignificant public health problem. However, it is classified as mild, moderate or severe when the prevalence of anemia is between 5.0 and 19.9%, 20.0 and 39.9%, or ≥40.0%, respectively (1).

Many studies have reported that the commonest cause of anemia in pregnancy is due to iron deficiency (14), especially in low- and middle-income countries (7), where it is attributed to poverty and malnutrition, more so among women and girls as a result of regular blood loss during menstruation. The demand for iron increases tremendously during pregnancy due to the increasing demand by the feto-placental unit as well as the need to compensate for blood loss during delivery. It is also usually the result of reduced intake or consumption of food rich in iron including red meat, green leafy vegetables (15, 16) as well as iron fortified food such as cereals and bread.

Anemia is defined by the presence of low hemoglobin (<11 g/dL), whereas iron deficiency is defined as low serum ferritin below 15 μg/L. Iron deficiency generally progresses in three stages from initial depletion of iron storage where the serum ferritin level begins to decline, through further depletion of iron storage with low serum ferritin and transferrin levels, and finally reduction in the hemoglobin level, at which point the iron deficiency is critically severe (17).

Besides iron deficiency, other causes of anemia include hemoglobinopathy, chronic diseases, hookworm infestation, chronic infections, and deficiencies of other nutritional elements such as vitamin B12 and folate (1). Identification and treatment of underlying causes of anemia is crucial (1) including encouraging iron supplementation during pregnancy especially among populations with increased prevalence of iron deficiency anemia (2).

The prevalence of antenatal anemia in Malaysia was first reported in 1997 as 47.5% (18), but the overall burden of anemia and its risk factors in pregnancy has not improved significantly since then. The contributory role of iron deficiency, or any other nutritional deficiency, to antenatal anemia in Malaysia has never been systematically appraised from published research. Identifying groups of women at high risk of anemia and iron deficiency in pregnancy will lead to more effective strategies to overcome this problem. With this in mind, we set out to perform a systematic review, the findings of which are anticipated to help plan interventional measures to alleviate the burden of antenatal iron deficiency anemia in this developing nation.

## Methods

### Study Design and Search Strategy

A systematic review of published original articles was conducted on the prevalence and determinants or risk factors of iron deficiency anemia among pregnant women in Malaysia. The review search was performed on databases that included Google Scholar, PubMed and Cochrane Library from the year 2000 until 2020. The search strategy followed the PICO strategy based on the title, abstract and MeSH terms.

The terms used for P (Population/Problem/Patient) were “pregnant women” OR “pregnant woman” OR “pregnant mother^*^” OR “antenatal” OR “pregnancy.” The terms used for I (Intervention/Issue) were “iron deficiency” OR “iron deficient” OR “iron deficiency anemia” OR “iron deficiency anemia” OR “hemoglobin” OR “hemoglobin.” The term used for C (Comparison) was “Malaysia.” The terms used for O (Outcome) were “risk factor^*^” OR “factor^*^” OR “determinant^*^” OR “cause^*^.” The Preferred Reporting Items for Systematic Reviews and Meta-Analysis (PRISMA) guideline was adhered to Moher et al. (19).

### Study Selection and Eligibility Criteria

We included published studies on risk factors of iron deficiency anemia among pregnant women in Malaysia. Studies were included if they were (1) conducted in health facilities or among communities in Malaysia; (2) in the form of original articles (not a protocol, review or commentary); (3) published between the years 2000–2020; and (4) published in the English language. There was no restriction in terms of minimum sample size.

**Figure 1:**
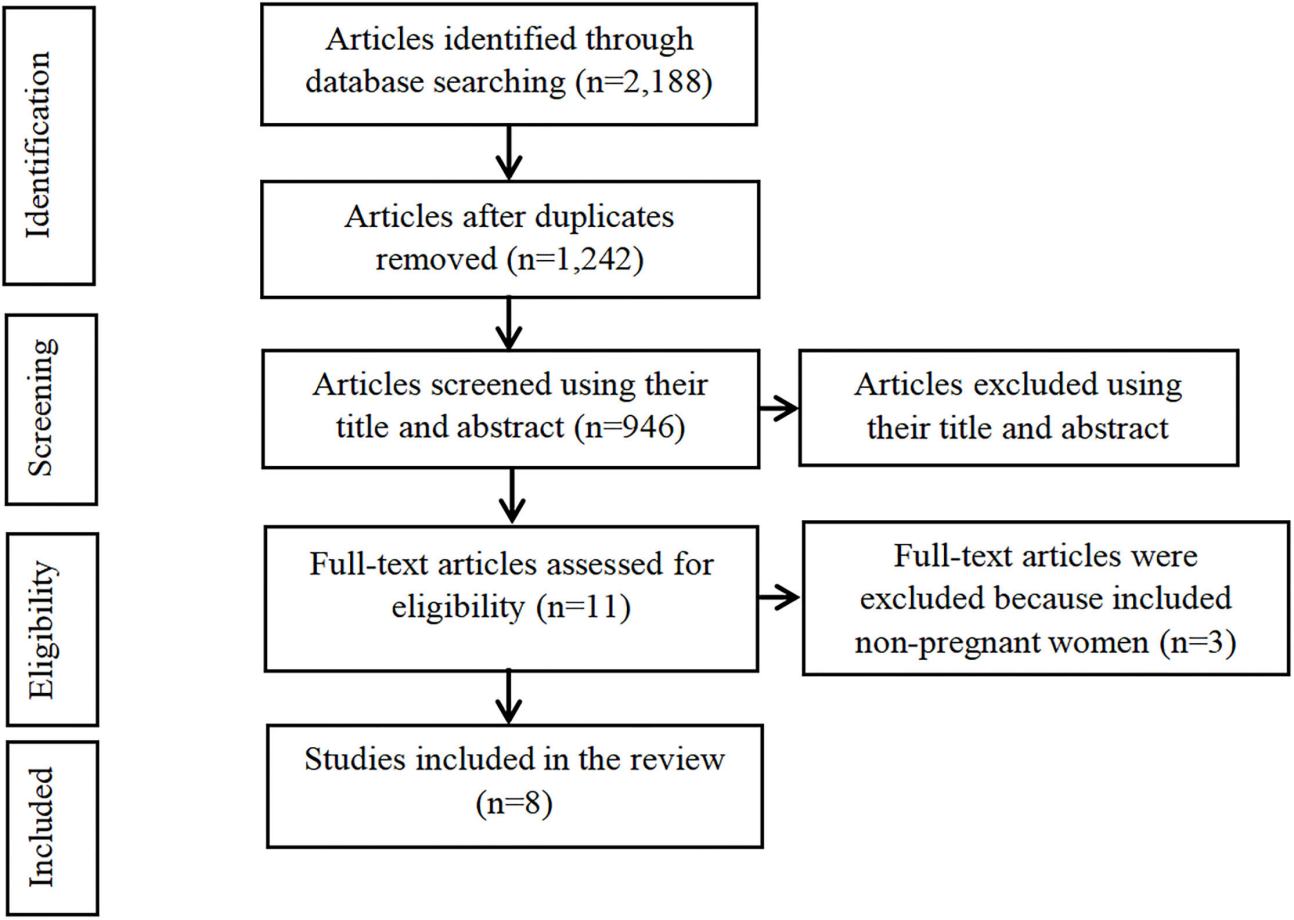
Flow diagram of the studies included in the systematic review.

Studies selected in the initial screening process were reviewed by two reviewers, who decided on the appropriateness of the study based on the title, abstract and keywords. Both reviewers must reach a consensus in order for the study to be accepted for the next phase of screening that involved retrieval of the full article for further scrutiny. A full text review of the articles was performed before they were included in the final review. The search flow is illustrated in Figure 1.

### Quality Assessment and Data Collection

We chose the Mixed Methods Appraisal Tool (MMAT) for quality assessment of the selected studies (20). For each selected publication, the sampling method was assessed for relevance to the research question, while the samples were assessed for representativeness of the target population, the measurement method for suitability, the level of risk for non-response bias, and the statistical analysis for appropriateness to answer the research questions (21). A standardized pre-designed data extraction table was used to capture all data for appraisal by the two reviewers, who also discussed articles with unclear information and finally assessed the overall quality.

## Results

### Study Selection

A total of 2,188 published articles were found on iron deficiency anemia among pregnant women in Malaysia. From this number, 1,242 duplicate papers were removed and 935 articles were excluded by screening the titles and abstracts. Eleven full-text articles were screened for eligibility. Subsequently, three articles were excluded for including non-pregnant women instead of pregnant women. Eight studies were finally included in this systematic review. Characteristics of the included studies are shown in Table 1.

**Table 1:** Characteristic of the included studies.

**No**.	**Authors, year of publication**	**Study location**	**Study design**	**Sample size**	**Prevalence of anemia in pregnancy *n* (%)**	**Risk factors for anemia**	**Adj OR, CI, *p*-value**	**Prevalence of ID/IDA/ID without anemia *n* (%)**	**Risk factors for iron deficiency**	**Risk of Bias**
1.	Rohim et al. (22)	Hulu Terengganu, Terengganu, Malaysia	Cross-sectional	182	33.2	Age (20–35 years old)	Adj OR 5.445 (95% CI 1.793, 2.149), *p* <0.001*	–	–	**+**
						Systolic BP (lower than 120 mmHg)	Adj OR, 1.225 (95% CI 0.402, 3.921), *p* <0.001*			
						Diastolic BP (lower than 90 mmHg)	Adj OR, 3.4 (95% CI 0.243, 1.129), *p* = 0.030*			
2.	Soh et al. (23)	Selangor, Malaysia	Cross-sectional	217	33.0	Educational level (lower educational level)	*p* = 0.001*	–	–	**++**
						Occupational (not working)	*p* = 0.01*			
						Family income (low family income)	*p* = 0.007*			
3.	Jusoh et al. (24) (Teenagers only)	Health clinics in north-western Malaysia	Cross-sectional	196	53.1	Late booking (>12 POA)	Adj OR, 16.33 (95% CI 6.51, 40.99), *p* <0.001*	–	–	**+**
4.	Nik Rosmawati et al. (25)	Kuala Besut Health Clinic, Jerteh, Terengganu, Malaysia	Cross-sectional	47	57.4	Hematinic compliance	Adj OR, 4.571 (95% CI 1.068, 19.573) *p* = 0.041*			**+**
5.	Mahdy et al. (26)	Health Clinic in urban area in a state in Malaysia	Cross-sectional	250	43.6	Race (Indian)	Mean (SD) serum ferritin, 27.17 ± 4.03, *p* <0.05*	79/250 (31.6)/52/250 (20.8)/27/79 (34.2)	Race (Indian)	**+**
						Parity (≥5)	Mean (SD) serum ferritin, 25.31 ± 3.99, *p* <0.05*		Parity (≥5)	
						Gestation age at booking (3rd trimester)	Mean (SD) serum ferritin, 19.95 ± 6.80, *p* <0.05*		Gestation age at booking (3rd trimester)	
						Primary Education	Mean (SD) serum ferritin		Primary Education	
						22.33 ± 7.85, *p* <0.05*		Primary Education	
6.	Hassan et al. (27)	Antenatal Clinic at Kubang Kerian, Kelantan, Malaysia	Cross-sectional	52	34.6	Serum ferritin was significantly associated with hemoglobin level	X2 (df), 8.54, *p* = 0.003*	18/52 (34.6)/11/52 (21.2)/7/18 (38.9)	–	**++**
7.	Tan et al. (28) (Primigravidae only)	Antenatal Clinic of University Medical Centre, Kuala Lumpur	Cross-sectional	622	19.3	Race (Indian)	*p* = 0.029*			**++**
8.	Haniff et al. (29)	56 health clinics nationwide	Cross-sectional	1,072	35.0	Second and Third trimester	Adj OR −1.41 (95% CI −1.69, −1.14), *p* <0.01*			**+**

Adj OR, Adjusted Odds Ratio; 95% CI, 95% Confidence Interval; x^2^, Chi-square, *significant at p < 0.05.

*RoB, Risk of Bias.*

*ID, Iron Deficiency.*

*IDA, Iron Deficiency Anemia*.

### Prevalence of Anemia in Pregnancy in Malaysia

The studies varied in design and focus. The overall prevalence of anemia in pregnancy ranged from 19.3 to 57.4%, while the prevalence of iron deficiency in pregnancy ranged from 31.6 to 34.6% (Table 1). Two studies conducted the research within specific populations i.e., among primigravidae only (28) and among teenagers only (24). The lowest prevalence of anemia (19.3%) was observed among primigravidae attending an urban university hospital in Kuala Lumpur (28). The highest prevalence (57.4%) was found among pregnant women attending a rural antenatal clinic in Terengganu, a state on the east coast of Malaysia (25).

### Risk Factors for Anemia and Iron Deficiency Anemia in Pregnancy

The risk factors that were significantly associated with antenatal anemia were younger age group (22), low family income (23), low educational level (23, 26) unemployment (23), late booking (24, 26), non-compliance to iron supplement (25), low serum ferritin level (27), being in the second or third trimester (26), and high parity (26, 29), as well as Indian ethnicity (26). Interestingly, one study found a significant relationship between anemia and low blood pressure (22).

In terms of ethnicity, a nationwide study revealed that hemoglobin was significantly lower among Malaysian Indian pregnant women with adjusted odds ratio (AOR) of 0.28 (95% CI 0.02, 0.54; *p* = 0.03) (29). Similarly, in another study that was conducted 6 years later, hemoglobin was also found to be significantly lower among Indian pregnant women, with mean (SD) of 11.57 (0.24) (*p* = 0.029) (28). An earlier study that was conducted by Tee et al. in 1984 among 309 women in the third trimester in the General Hospital, Kuala Lumpur, concurred that anemia was highest among Indian mothers (30).

A study that specifically evaluated determinants of iron deficiency among pregnant women revealed that serum ferritin level was significantly lower among mothers who were Indian, grand multiparous, and booked late for antenatal care (26). Another study that addressed the issue of poor compliance to hematinics among pregnant women in a rural area of Terengganu showed that the prevalence of non-compliance was as high as 36.2% (25). The prevalence of anemia within the non-compliant group was 76.5% compared to 57.4% overall.

## Discussion

We believe that this is the first systematic review on anemia and iron deficiency among pregnant women in Malaysia. From this review we found that there was very little focus on IDA or any other specific nutritional deficiency as a cause of anemia. The quality and format of reporting of the publications that were finally chosen were variable. Out of the final selection of eight articles, only two reported objectively on iron deficiency and iron deficiency anemia (26, 27) through assessment of serum ferritin. In view of the extremely limited number of publications on prevalence and risk factors of iron deficiency, articles on anemia in pregnancy in general that do not specify iron deficiency anemia were also included in this review.

Our systematic review revealed that the prevalence of anemia in pregnancy in Malaysia ranged between 19.3 and 57.4%, whereas the prevalence of iron deficiency anemia was 20.8 to 21.2%. The prevalence of anemia in pregnancy is comparable to countries with poorer economy, as the pooled prevalence of anemia among pregnant women in Ethiopia was 31.66% (31) and in Sudan, 53.0% (32). However, the prevalence of anemia among pregnant women in Iran, a comparable developing nation, was 13.6%, which was much lower (33). The two papers (26, 27) that included assessment of serum ferritin reported similar findings in terms of a clinically significant proportion of pregnant women with iron deficiency who had normal hemoglobin levels (34.2 and 38.9%, respectively).

It is vital for the Malaysian health services and authorities to delve deeper into this problem in order to find the root cause and rectify the problem. Anemia in pregnancy has long been a neglected cause that does not garner as much attention as non-communicable diseases and cancer. Nonetheless, it is time to realize that mitigating this problem is as impactful as the other areas of focus. Irrespective of whether they are home-makers or in the workforce, anemia among pregnant women leads to decline in productivity primarily by affecting maternal cognitive function and attention (34). Moreover, reducing the prevalence of anemia by 1 g% reduces the risk of maternal death by 29% (35). Anemia in pregnancy can also potentially affect the next generation in many ways, including increasing the risk of adult hypertension in later life, and adversely affecting cognitive function and behavior (36). Juvenile delinquency and youth crime rates are known to be rampant among certain socio-economic and ethnic groups, and it will be interesting to study whether this is the result of exposure *in utero* to maternal anemia (37).

The prevalence of anemia in pregnancy was noted to be generally higher in rural areas i.e., 53.1% in north-west Malaysia (24) and 57.4% in Terengganu (25), compared to 19.3% in a university hospital in Kuala Lumpur, the capital city of Malaysia (28), 33.0% in an urban area in Selangor (23), and 43.6% in another urban area on the west coast (26). These findings were similar to another systematic review conducted in Ethiopia where pregnant women residing in urban areas were 73% less likely to be anemic (31). This may be related to better information and educational access in urban areas (38), hence leading to better health awareness, and a healthier diet and lifestyle. It may also be related to the affluence of urban communities (39). However, the urban poor may do worse than rural communities, as evidenced by findings in the nationwide study by Haniff et al. (29).

**Figure 2:**
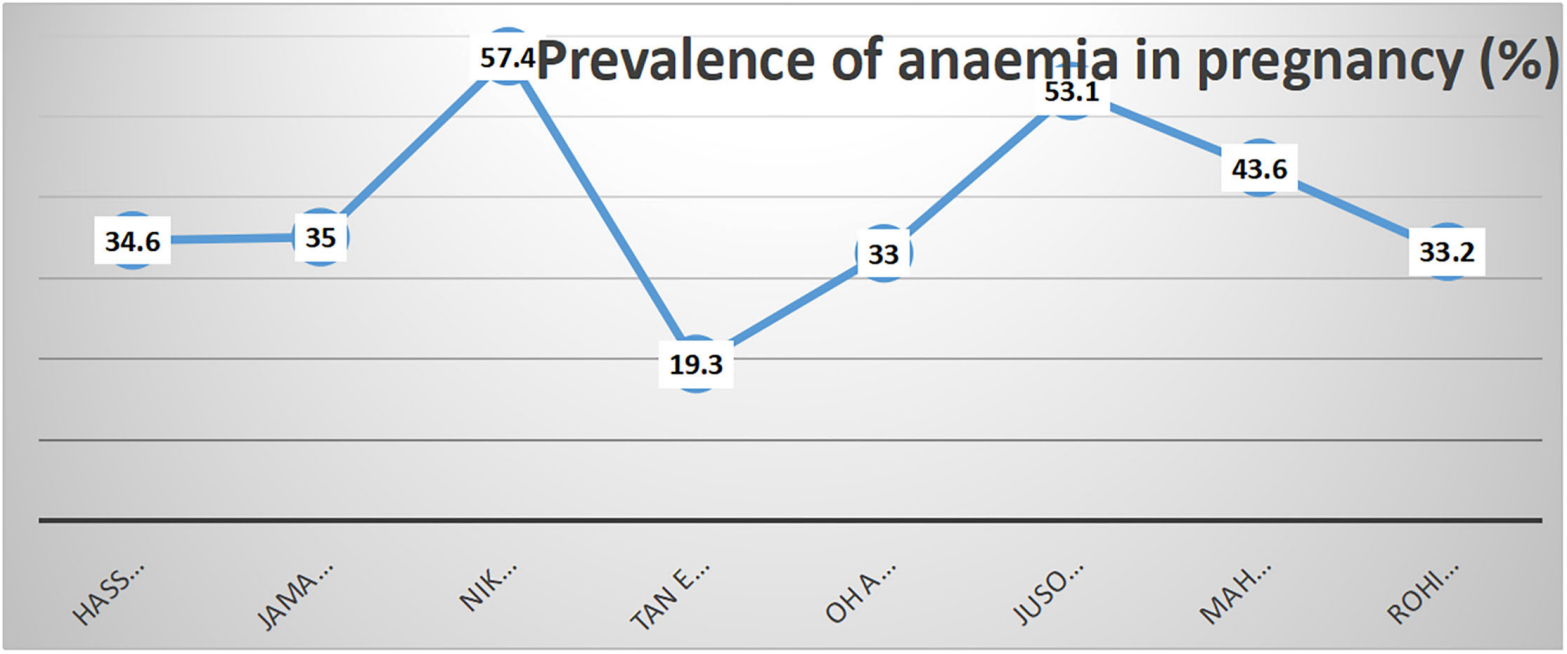
Prevalence of anemia in included studies in sequence according to year of publication.

One striking discovery was the absence of any truly progressive improvement in the prevalence of anemia in pregnancy in Malaysia over a span of 11 years, i.e., more than a decade (Figure 2). The study by Haniff et al. (29) covering 59 government health clinics nationwide reported a prevalence of 35.0% in 2007 (27), whereas the most recently reported prevalence by Rohim et al. (22) was 33.2%. This again raises the question of whether enough has been done over the years to curb this outstanding national problem.

The reported risk factors appear to follow a similar pattern throughout all selected articles. Risk factors for anemia in pregnancy in general were younger maternal age (22), late antenatal booking (24), non-compliance toward hematinics (25), being in the second or third trimester (29), lower educational level, unemployment, and low family income (23). Meanwhile, Mahdy et al. (26) singled out Indian ethnicity, grandmultiparity, late antenatal booking and low educational status as factors that were significantly associated with antenatal iron deficiency anemia. The higher prevalence of anemia among Indians is probably attributable to poor socio-economic status, low educational level, and dietary habit (as some are vegetarian due to cultural or religious reasons), hence B12 deficiency may also contribute. The first two factors may consequently influence their access to health services and reduce awareness of the importance of early booking in pregnancy in order to receive appropriate care including iron supplementation. One study (Mahdy et al.) revealed that late booking of pregnancy and low educational level were also independent risk factors for iron deficiency anemia, however no cross analysis was performed to link the high prevalence of iron deficiency anemia among Indian mothers to low educational level and late booking (26).

One article focused only on teenage pregnancies (24), whereas another recruited only primigravidae, giving a clearer perspective into these two important groups, but disallowing age and parity comparisons (28). In the study by Jusoh et al. (24), the prevalence of anemia among pregnant teenagers was shockingly high, and among the contributory factors was the unmarried status of these mothers, hence suggesting poor family support, particularly financial and social support. The study by Tan et al. (28) focused on primigravidae only, revealing the lowest prevalence of anemia in the series, and this further confirms the role of increasing parity on the risk of anemia.

A systematic review on determinants of anemia in pregnancy in Ethiopia revealed that multiparity was a risk factor, with primigravidae being 61% less likely to become anemic (31). Contrary to these findings, a systematic review from Sudan revealed that age and parity were not significantly different between anemic and non-anemic pregnant women. Anemia in pregnancy was prevalent in Sudan regardless of age and parity (32). Meanwhile, in Kenya, risk factors associated with anemia in pregnancy were low socioeconomic status, low educational level, and being in the second or third trimester (40). Similar to our systematic review, the risk factors that were associated with anemia among pregnant women in Indonesia were non-compliance to hematinic and low educational level (41).

Out of the eight publications, the only study that looked into compliance to iron supplementation was conducted by Nik Rosmawati et al. (25), which looked into anemia in pregnancy among a rural population in the state of Terengganu on the East Coast of Malaysia. The findings highlight the importance of managing patient compliance in any endeavor to improve the iron status of pregnant women. In order to achieve good patient compliance, we believe it is crucial to empower the patient with adequate knowledge on the do's and don'ts of iron consumption, besides emphasis on a good diet that is rich in iron, folate and vitamin B12.

An interesting observation by Rohim et al. (22) was that low blood pressure was significantly associated with low hemoglobin. This is likely to be attributable to hemoconcentration and hence significantly higher ferritin concentration that is expected among women with preeclampsia manifesting with raised blood pressure (42). Therefore, low blood pressure is not an appropriate risk factor to be included as a valid feature of the target group for remedial measures to overcome anemia in pregnancy.

With regard to prevention of iron deficiency anemia in pregnancy, Malaysia like most developing countries, practices the preventive approach by prescribing iron supplements routinely to all pregnant women (2). Despite the iron supplementation, there is not much improvement in the prevalence of anemia in pregnancy (2). The main obstacle for this problem is poor compliance to the iron supplement (34). Compliance is important to ensure adequate iron storage and to ensure improvement in the maternal iron status (8). Mothers are provided with supplementation according to each clinic or hospital's own protocol without proper monitoring of compliance, which is a personal choice. However, about half of the pregnant women in Malaysia do not consume the iron supplements due to forgetfulness, intolerance to side effects of iron tablets and the mythical fear of having big infants as a result of iron tablets consumption (43). Nevertheless, other causes of nutritional anemia such as folate and Vitamin B12 deficiencies should also be given due consideration in preventing anemia in pregnancy.

### Strengths and Limitations

We believe this is the first attempt at systematically reviewing articles on the subject of antenatal anemia and iron deficiency in Malaysia. An obvious limitation is the small number of publications on iron deficiency among pregnant women in Malaysia and the substantial variation from each other in terms of the parameters assessed. This limits the comparability among the reported studies. Our review is also limited by the exclusion of unpublished conference papers and gray literature, and publications in languages other than English.

### Framework for Future Intervention

A well-planned epidemiological study on both anemia and iron deficiency among pregnant women in Malaysia is urgently required in order to look into the current situation and rectify the problem (44). Future studies should also include investigation into other nutritional causes of anemia among pregnant women so that appropriate recommendations can be made to solve the problem. In the meantime, based on the current data as reviewed, remedial measures should be focused on target groups that are identifiable from our systematic review, i.e., Indian mothers, women at the extremes of childbearing age, multiparous women, communities with low socioeconomic and education level, and mothers who book late for antenatal care. Teenage pregnancy, especially single mothers, is another group that requires attention. Dietary iron intake is not sufficient to overcome anemia in pregnancy thus oral iron supplements should be included in the management (45). Compliance to oral iron supplements is vital, hence this area must be explored in terms of prediction of non-compliance and measures to overcome, such as patient education. A previous study mentioned that compliance to vitamins and mineral supplements in Malaysia is considerably low i.e., around 49% (43).

## Conclusion

The overall prevalence of anemia among pregnant women in Malaysia ranged from 19.3 to 57.4%, whereas the prevalence of iron deficiency was 31.6 to 34.6%. Because of the high prevalence of anemia and iron deficiency among pregnant women in Malaysia, these are important and major public health problems due to the adverse effects to both mother and fetus. The risk factors for anemia in pregnancy in Malaysia were extremes of reproductive age, grandmultiparity, unmarried teenage parenthood, Indian ethnicity, low educational level, low socioeconomic status, late antenatal booking, rural residence (and urban poor) and non-compliance to iron tablets. Measures to remedy this important problem should focus on these high-risk groups by introducing a good public health audit mechanism.

## Author Contributions

ZM conceptualized the systematic review. RauA and II performed literature search, screening process to include studies that met inclusion criteria and quality assessment of the included studies, and prepared the manuscript. II, ZI, and RahA reviewed the manuscript. ZM reviewed the final version of manuscript. All authors contributed to the article and approved the submitted version.

## Conflict of Interest

The authors declare that the research was conducted in the absence of any commercial or financial relationships that could be construed as a potential conflict of interest.

## Publisher's Note

All claims expressed in this article are solely those of the authors and do not necessarily represent those of their affiliated organizations, or those of the publisher, the editors and the reviewers. Any product that may be evaluated in this article, or claim that may be made by its manufacturer, is not guaranteed or endorsed by the publisher.
